# A new insight into the apoptotic effect of nitidine chloride targeting Checkpoint kinase 2 in human cervical cancer *in vitro*

**DOI:** 10.3164/jcbn.19-28

**Published:** 2019-10-08

**Authors:** Hye-Jeong Kwon, Lee-Han Kim, Chi-Hyun Ahn, In-Hyoung Yang, Kyoung-Ok Hong, Seong Doo Hong, Ji-Ae Shin, Sung-Dae Cho

**Affiliations:** 1 Department of Oral Pathology, School of Dentistry and Dental Research Institute, Seoul National University, 101 Daehak-ro, Jongno-gu, Seoul 03080, Republic of Korea; 2 Department of Microbiology, Institute for Immunology and Immunological Diseases, Yonsei University College of Medicine, 50-1 Yonsei-ro, Seodaemun-gu, Seoul 03722, Republic of Korea

**Keywords:** cervical cancer, nitidine chloride, Chk2 activation, apoptosis

## Abstract

Nitidine chloride (NC), a natural, bioactive, phytochemical alkaloid derived from the roots of *Zanthoxylum nitidum*, has been reported to exhibit anti-tumor activity against various types of cancer. However, the potential therapeutic role of NC in human cervical cancer has not yet been studied. We are the first to report that NC acts as a potential apoptosis-inducing agent for human cervical cancer *in vitro*. NC treatment of human cervical cancer cell lines induced caspase-mediated apoptosis, thereby reducing cell viability. Phospho-kinase proteome profiling using a human phospho-kinase array revealed that NC treatment phosphorylated Checkpoint kinase 2 (Chk2) at Thr68, which activates Chk2 in both cell lines. We also found that NC significantly affected the p53/Bim signaling axis, which was accompanied by mitochondrial membrane depolarization and cytochrome c release from the mitochondria into the cytosol. In addition, NC profoundly increased phosphorylation of the histone variant H2AX at Ser139, a typical marker of DNA damage. Taken together, these results provide *in vitro* evidence that NC can increase Chk2 activation, thereby acting as an attractive cell death inducer for treatment of human cervical cancer.

## Introduction

Nitidine chloride (NC) is a natural, bioactive, chemical alkaloid isolated from the roots of *Zanthoxylum nitidum*, a traditional herbal medicine. Previous studies have shown that NC inhibits the production of various cytokines by modulating the nuclear factor kappa-B (NF-κB) and mitogen-activated protein kinase signaling pathways in lipopolysaccharide-stimulated RAW 264.7 macrophage cells,^(1)^ and that it suppresses receptor activator NF-κB ligand-induced osteoclastogenesis *in vitro* and osteolysis *in vivo*.^(2)^ In cancer biology, NC showed potent anti-tumor properties in a wide range of cancers by inhibiting cell proliferation and inducing cell cycle arrest or apoptosis through dephosphorylation of Akt or extracellular signal-regulated kinase.^(3–5)^ It has also been found that the inhibitory effect of NC on the signaling pathway of signal transducer and activator of transcription 3 (STAT3) contributes to inhibition of neovascularization and tumorigenesis by vascular endothelial growth factor.^(6,7)^ Recently, our team found that the biological action of NC plays a crucial role in inducing apoptosis through inhibition of p-STAT3 or myeloid cell leukemia-1 in oral cancer.^(8,9)^ However, despite a variety of studies on the potential biological role of NC, it remains unclear whether it has potential as a chemotherapeutic drug candidate for cervical cancer treatment.

Checkpoint kinase 2 (Chk2), an essential signal transducer upon DNA damage, is mainly phosphorylated and activated by ataxia telangiectasia mutated serine/threonine kinase (ATM) on the priming site Thr68, which leads to phosphorylation of cellular substrates involved in DNA repair and apoptosis.^(10,11)^ In addition to the role of Chk2 in DNA damage, Chk2 activation is necessary to maintain chromosomal stability by phosphorylating the tumor suppressor Braca1 during mitosis.^(12)^ The loss of *Chk2* by mutation, deletion, or epigenetic silencing has been associated with risk of tumor formation and chemoresistance,^(12–14)^ suggesting that Chk2 is a tumor suppressor. Accumulated data suggest that natural compounds can increase Chk2 phosphorylation, resulting in DNA damage–induced apoptosis or G2 phase arrest, apparently through inhibition of topoisomerase Ⅱ activity.^(15,16)^ Several natural compounds were associated with cell cycle arrest via the activated ATM/Chk2 signaling pathway in the presence or absence of p53 activation.^(17,18)^ Therefore, it is worthwhile to investigate the correlation between NC and Chk2 in cervical cancer.

In the present study, we clarified the mechanism underlying the apoptotic activity of NC *in vitro* and explored the potential of NC as an anticancer drug candidate for cervical cancer.

## Materials and Methods

### Cell culture and chemical treatment

HEp-2 and KB cells were obtained from Kyungpook National University (Daegu, Korea) and the American Type Culture Collection (Manassas, VA), respectively. Cells were cultured in DMEM/F-12 medium supplemented with 10% fetal bovine serum (FBS) for HEp-2 cells or 5% FBS for KB cells and 1% penicillin/streptomycin in a 5% CO_2_ incubator at 37°C. When the cells reached 50–60% confluence, they were treated with NC (Sigma-Aldrich, St. Louis, MO) dissolved in dimethyl sulfoxide (DMSO). The final concentration of DMSO did not exceed 0.1%.

### Measurement of cell viability

The trypan blue exclusion assay was used to measure the effect of NC on cell viability. Cells were stained with 0.4% trypan blue solution (Gibco, Paisley, UK), and viable cells were counted with a hemocytometer. All experiments were performed three times, with triplicates in each independent experiment.

### Live/dead assay

The cytotoxicity of NC was examined using a Live/Dead & Viability/Cytotoxicity Kit (Life Technologies, Grand Island, NY). Briefly, cells were stained with 2 µM Calcein-AM and 4 µM ethidium homodimer-1 and then incubated for 30 min at room temperature (RT). Live (green fluorescence) and dead (red fluorescence) cells were visualized under a fluorescence microscope (Leica DMi8, Wetzlar, Germany) with appropriate excitation and emission filters.

### 4'-6-Diamidino-2-phenylindole staining

Cells were stained with 4'-6-diamidino-2-phenylindole (DAPI) solution (Sigma-Aldrich) to confirm the presence of nuclear morphological changes associated with apoptotic cells. Briefly, cells were fixed in 100% ethanol overnight at −20°C, deposited on slides, and stained with DAPI fluorescent dye (2 µg/ml). A fluorescence microscope was used to observe the morphological characteristics of apoptotic cells: nuclear condensation and fragmentation.

### Western blotting

Whole cell lysates were extracted with RIPA lysis buffer (EMD Millipore, Billerica, CA) containing phosphatase inhibitor and protease inhibitor cocktail. Protein concentrations of whole cell lysates were measured using a DC Protein Assay Kit (Bio-Rad Laboratories, Madison, WI). After normalization, equal amounts of protein were separated by sodium dodecyl sulfate-polyacrylamide gel electrophoresis and transferred to Immuno-Blot PVDF membranes. The membranes were blocked with 5% skim milk in Tris-buffered saline with Tween20 for 2 h at RT, incubated with specific primary antibody overnight at 4°C, and finally probed with horseradish peroxidase (HRP)-conjugated secondary antibody for 2 h at RT. Antibodies that detect cleaved caspase 3, cleaved poly(ADP-ribose) polymerase (PARP), p-Chk2 (Thr68), Chk2, γH2AX, H2AX, Bim, Bax, and Puma were purchased from Cell Signaling Technology, Inc. (Charlottesville, VA). Actin and α-tubulin antibody were obtained from Santa Cruz Biotechnology, Inc. (Santa Cruz, CA). COX4 antibody was purchased from Abcam (Cambridge, UK). Antibodies against cytochrome c and p53 were obtained from BD Biosciences (San Diego, CA) and Calbiochem (San Diego, CA), respectively. Protein bands were immunoreacted with ECL solution (Santa Cruz Biotechnology, Inc., Santa Cruz, CA) and then visualized by an ImageQuant LAS 500 (GE Healthcare Life Sciences, Piscataway, NJ) or X-ray film.

### Human phospho-kinase proteome profiling

Cells were treated with 10 µM NC for 3 h, and then phospho-kinase proteome profiling was performed using a Human Phospho-Kinase Array Kit (R&D Systems, Minneapolis, MN), which can detect the phosphorylation levels of 43 kinases, according to the manufacturer’s protocol. Briefly, the membrane was blocked with array buffer for 1 h at RT. The cell lysate was then added and kept on a rocking platform shaker overnight at 4°C. After washing with 1× washing buffer, the membrane was incubated with antibody cocktails at RT for 2 h and then incubated with streptavidin-HRP for 30 min at RT. Capture spots corresponding to the amount of kinase protein bound were detected with 1 ml of Chemi reagent mix.

### Immunofluorescence staining

Cells were seeded on 4-well culture plates and treated with DMSO or 10 µM NC. After NC treatment, cells were fixed and permeabilized with cytofix/cytoperm solution (BD Bioscience, San Diego, CA) for 1 h at 4°C. Cells were blocked with 1% bovine serum albumin in phosphate buffered saline (PBS) for 1 h at RT and incubated overnight at 4°C with antibodies against p-Chk2 (Thr68) or γH2AX. Cells were then exposed to fluorescein-5-isothiocyanate (FITC)-conjugated secondary antibody for 1 h at RT and visualized using a fluorescence microscope equipped with filters suitable for DAPI and FITC dyes.

### Preparation of cytosolic and mitochondrial fractions

Cytosolic and mitochondrial fractions were isolated using a Mitochondria/Cytosol Fractionation Kit (Abcam). Briefly, cells were washed with ice-cold PBS, and the cell pellet was resuspended in 1× cytosol extraction buffer mix containing DTT and protease inhibitor for 10 min on ice. After centrifugation at 13,000 rpm for 15 min at 4°C, the supernatants containing the cytosolic proteins were collected, and the pellets were resuspended in mitochondrial extraction buffer mix. The supernatant containing mitochondrial proteins was collected from a final centrifugation.

### Mitochondrial membrane potential assay

 Mitochondrial membrane potential (ΔΨm) was measured by flow cytometry using a lipophilic fluorescent dye, JC-1 (BD Biosciences). Cells were harvested by trypsinization, washed with PBS, and then pelleted by centrifugation at 3,500 rpm for 5 min. The pellets were resuspended in 1× JC-1 working solution and incubated at 37°C for 30 min in the dark. The stained cells were washed with 1× assay buffer and collected by centrifugation at 3,500 rpm for 5 min. After removing the supernatant, they were resuspended in 1× assay buffer. Cells were then transferred to FACS tubes and analyzed with FACSCalibur (BD Biosciences). At least 10,000 events per sample were counted.

### Statistical analysis

All statistical analyses of the results were performed using SPSS ver. 22.0 (SPSS Inc. Chicago, IL). One-way ANOVA with Tukey’s post hoc test was applied to multiple comparisons to determine the significance of differences between the control and treatment groups. A *p* value less than 0.05 was considered statistically significant.

## Results

### NC produces cytotoxic effects by inducing caspase-mediated apoptosis in human cervical cancer cell lines

**Fig. 1:**
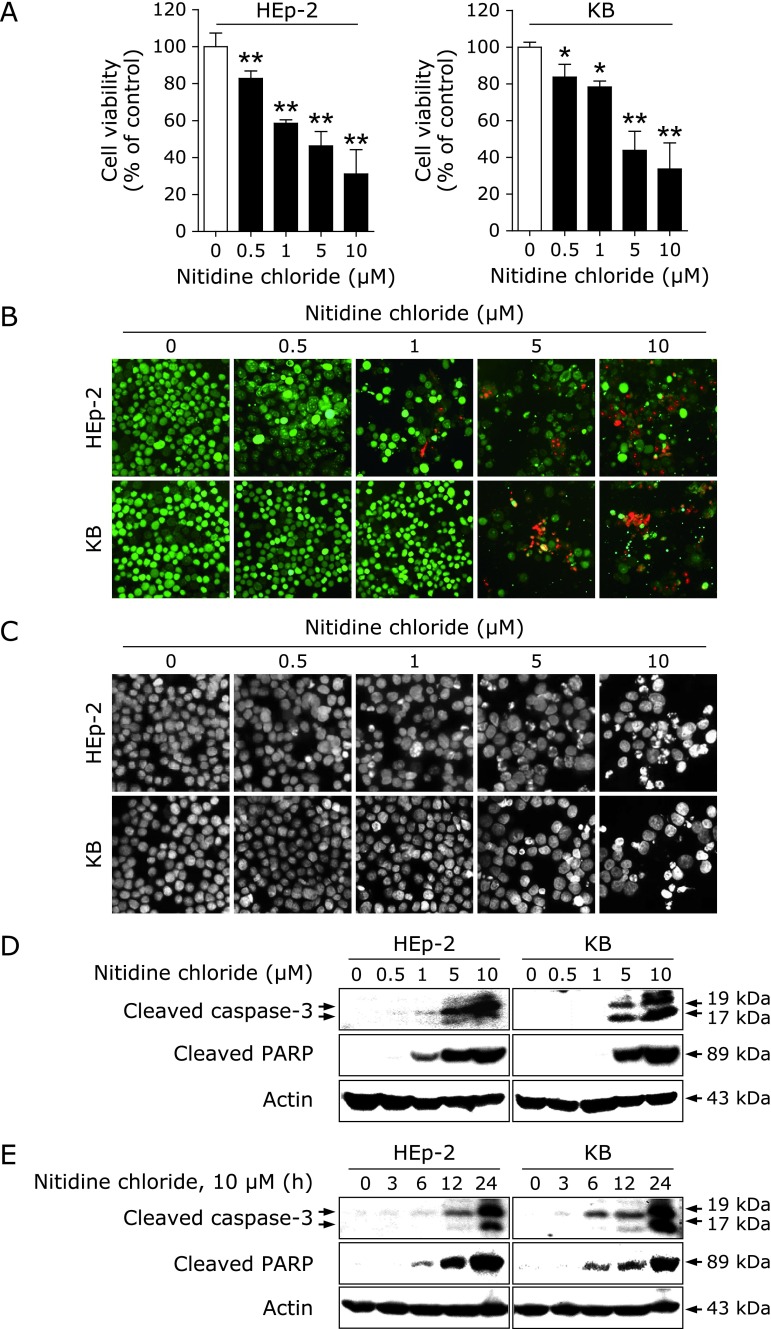
Effects of NC on cell viability and apoptosis in human cervical cancer cell lines. HEp-2 and KB cells were treated with DMSO or the indicated concentration of NC (0.5, 1, 5, or 10 µM) for 24 h. (A) Cell viability was evaluated using a trypan blue exclusion assay. The graphs express the mean ± SD of triplicate experiments (*p<0.01, **p<0.001). (B) Live (green fluorescence) and dead (red fluorescence) cells were observed under a fluorescence microscope. Representative images are displayed (magnification, ×200). (C) Cells stained with DAPI solution were observed under a fluorescence microscope. Representative images are displayed (magnification, ×400). (D, E) Protein levels of cleaved caspase-3 and PARP were determined by Western blotting. Actin was used as a loading control.

To investigate the cytotoxic effect of NC on human cervical cancer cell lines, trypan blue exclusion assays were performed in HEp-2 and KB cell lines treated with NC at concentrations of 0.5–10 µM for 24 h. As shown in Fig. 1A, NC significantly reduced the viability of both cell lines, with IC_50_ values of 3.9 µM and 4.7 µM, respectively. The live/dead assay showed an increase in cells stained with red fluorescence, suggesting membrane-damaged dead cells (Fig. 1B). To investigate the possible potential toxicity of NC, we also monitored the effect of NC on cell viability of human oral keratinocyte (HOK). As shown in Supplemental Fig. 1*, NC has less growth-inhibitory effect on HOK compared to two cervical cancer cell lines. To investigate whether the NC-induced cytotoxic effect was due to apoptosis, we looked for characteristics of apoptosis such as chromatin condensation and DNA fragmentation using DAPI, a fluorescent DNA-binding dye. Those results showed an increase in apoptosis in the NC-treated cells compared with DMSO-treated cells (Fig. 1C). We also found that NC increased the cleavage of caspase-3 or PARP, an apoptotic index, in a concentration- and time-dependent manner (Fig. 1D and E). These results suggest that the cytotoxic effect of NC in human cervical cancer cell lines might be related to caspase-mediated apoptosis.

### NC causes Chk2 activation by phosphorylating Thr68 in human cervical cancer cell lines

**Fig. 2:**
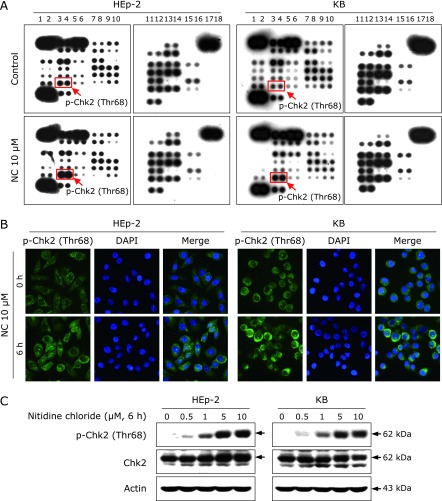
Effect of NC on Chk2 activation in human cervical cancer cell lines. (A) HEp-2 and KB cells were treated with DMSO or 10 µM NC for 3 h, and the cell lysates were analyzed using the human phospho-kinase array. (B) Immunofluorescence staining of p-Chk2 (Thr68) in HEp-2 and KB cells treated with NC for 6 h. Representative images of staining for p-Chk2 (Thr68, green) and counterstaining with DAPI (blue) are shown. The merge panel combines the two images (magnification, ×400). (C) HEp-2 and KB cells were treated with the indicated concentrations of NC for 6 h. Protein levels of p-Chk2 (Thr68) and Chk2 were determined by Western blotting.

To explore the mechanism of NC-induced apoptotic activity, we analyzed the phosphorylation profiles of 43 kinases using a human phospho-kinase array. As shown in Fig. 2A, NC treatment markedly induced phosphorylation of Chk2 at Thr68 compared with the control group. To confirm that result, we performed immunofluorescence staining using a p-Chk2 antibody specific to its activation site, Thr68. We found that the phosphorylation status of Chk2 increased in both cell lines after 6 h of NC treatment (Fig. 2B). Consistent with these results, western blot analysis showed that NC treatment induced high expression of p-Chk2 (Thr68) in a concentration-dependent manner (Fig. 2C). In contrast, we did not find an increase in total Chk2 in NC-treated cells. Thus, phosphorylation of the Thr68 site might be necessary for NC-induced cell death in human cervical cancer cell lines.

### NC affects the p53/Bim signaling pathway in human cervical cancer cell lines

**Fig. 3:**
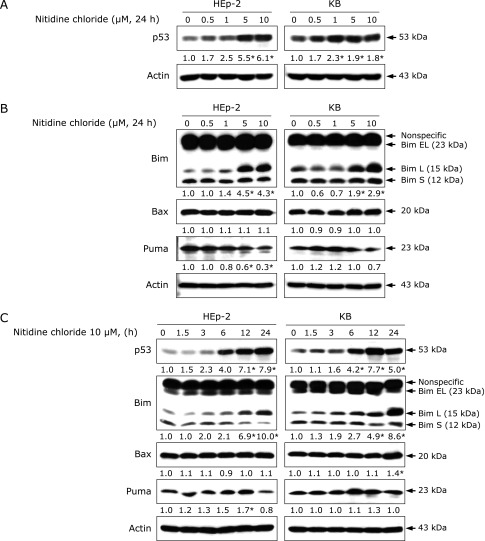
The effects of NC on p53 and its downstream targets in human cervical cancer cell lines. (A, B) HEp-2 and KB cells were treated with DMSO or the indicated concentration of NC for 24 h. Protein levels of p53, Bim, Bax, and Puma were analyzed by western blotting. (C) HEp-2 and KB cells were treated with 10 µM NC for the indicated time points. Levels of p53, Bim, Bax, and Puma proteins were detected by western blotting. Values represent the mean of three independent experiments (*p<0.05).

Next, we used western blotting to assess the expression level of p53 to clarify the association between p53 and Chk2-related apoptosis induced by NC. NC treatment resulted in a significant increase in p53 protein expression in both cell lines (Fig. 3A). We investigated three Bcl-2 family proteins (Bim, Bax, and Puma) that are downstream molecules of the p53 protein, and we found an increase in Bim protein expression after NC treatment. The protein expression patterns of Bax and Puma did not increase significantly (Fig. 3B). NC increased p53 and Bim at similar time points in both cell lines after treatment for 6–24 h, but minimal effects were observed before that (Fig. 3C). Thus, NC might induce p53/Bim signaling pathways that could be involved in NC-induced apoptosis in human cervical cancer cell lines.

### NC leads to loss of ΔΨm, which causes cytochrome c release into the cytosol in human cervical cancer cell lines

**Fig. 4:**
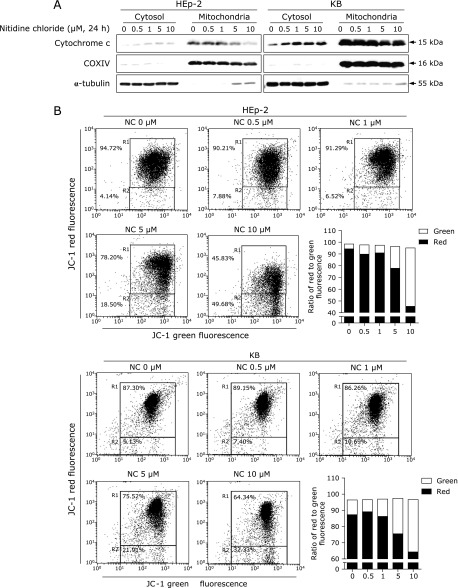
The effects of NC on ΔΨm and cytochrome c release in human cervical cancer cell lines. (A) Cytochrome c release into the cytosol was assessed using cytosolic and mitochondrial fractions. COX IV and α-tubulin were used as specific markers for mitochondria and cytosol, respectively. The experiments were performed twice. (B) The effect of NC on ΔΨm was evaluated by JC-1 staining. A representative R1 (upper region)/R2 (lower region) profile with red/green fluorescence is shown. The upper region represents healthy cells with normal ΔΨm, and the lower region represents apoptotic cells with depolarized ΔΨm. Bar graphs show the ratio of red to green fluorescence. The experiments were performed twice.

Because our data showed that NC induced the p53/Bim signaling pathway and caspase-3 activation, we assumed that, during NC treatment, the p53/Bim signaling pathway regulates mitochondrial membrane depolarization, which would cause an efflux of cytochrome c from mitochondria. As shown in Fig. 4A, cytochrome c was released from mitochondria into the cytosol in both cell lines in response to NC treatment. We next observed ΔΨm using JC-1, a fluorescence-based, mitochondria-specific dye that aggregates in the mitochondria of healthy cells (red fluorescence) and is present as a monomer in the cytosol in the presence of apoptotic signals (green fluorescence). The results show that the proportion of cells with loss of ΔΨm was increased by NC treatment (Fig. 4B), suggesting that NC induced apoptosis through mitochondrial membrane depolarization.

### NC enhances the accumulation of γH2AX in human cervical cancer cell lines

**Fig. 5:**
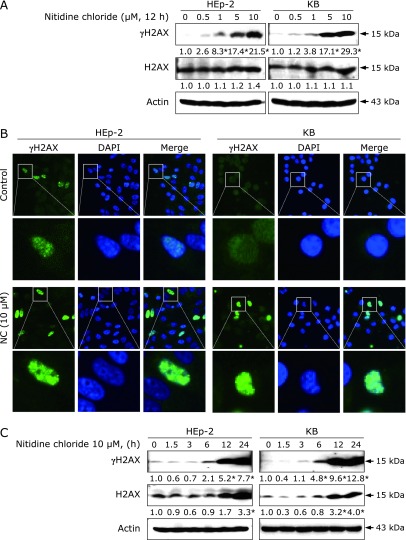
Effect of NC on γH2AX accumulation in human cervical cancer cell lines. HEp-2 and KB cells were treated with DMSO or the indicated concentration of NC for 12 h. (A) The expression levels of γH2AX and H2AX were detected using western blotting. Values represent the mean of three independent experiments (*p<0.05). (B) Immunofluorescence staining of γH2AX in HEp-2 and KB cells. Representative images of the staining for γH2AX (green) and counterstaining with DAPI (blue) are shown. The merge panel combines the two images (magnification, ×400). Enlarged views of the white line box areas are shown (lower row) for better visualization of γH2AX foci. (C) HEp-2 and KB cells were treated with 10 µM NC for the indicated times. Levels of γH2AX and H2AX were detected by western blotting. Values represent the mean of three independent experiments (*p<0.05).

**Fig. 6:**
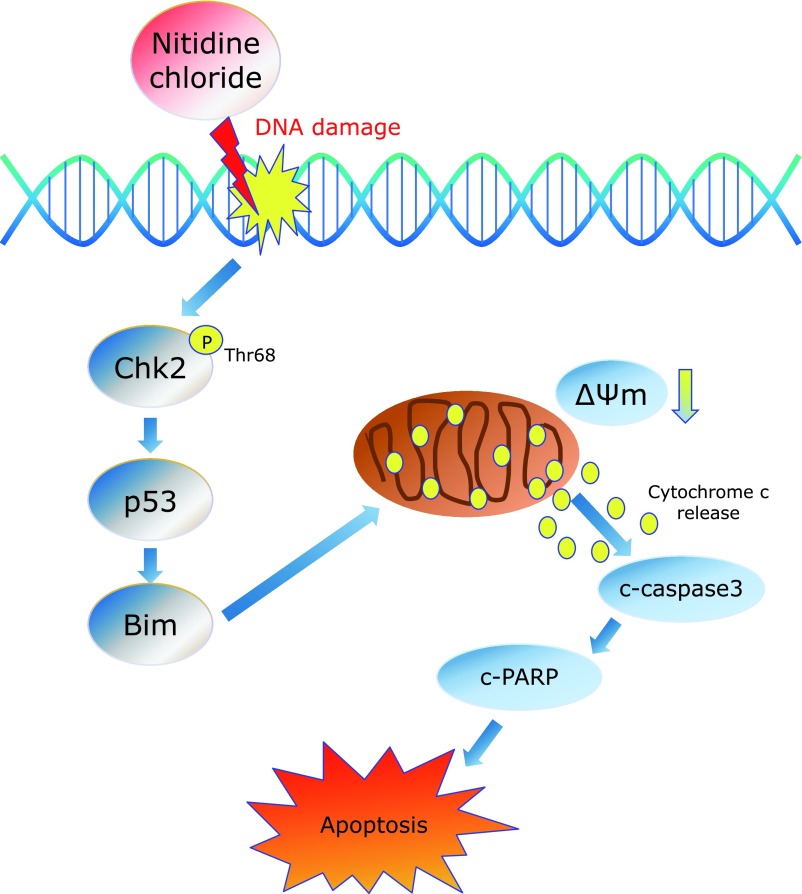
A proposed working model of the molecular mechanisms underlying the pro-apoptotic action of NC through Chk2 activation in human cervical cancer cell lines. NC-induced apoptosis is mediated by Chk2 activation, which promotes p53/Bim signaling that leads to depolarization of mitochondrial membrane potential.

Because the DNA damage response can lead to induction of apoptosis, we investigated the possibility that NC elicits the phosphorylation of histone variant H2AX at Ser139 (called γH2AX), a typical marker used to examine DNA damage. As shown in Fig. 5A, NC significantly increased γH2AX in a concentration-dependent manner, but we found no significant changes in total H2AX level. To further assess the formation of γH2AX in response to DNA damage after NC treatment, we quantified γH2AX foci in the nucleus and found that NC increased the number of γH2AX foci in the nucleus (Fig. 5B). We also observed that γH2AX accumulated in a time-dependent manner after NC treatment (Fig. 5C). Therefore, the apoptotic effect of NC in human cervical cancer cell lines might somehow be a consequence of the accumulation of γH2AX. A summary of the working model by which NC has pro-apoptotic action through Chk2 activation in human cervical cancer cell lines is illustrated in Fig. 6.

## Discussion

Cervical cancer is the fourth most frequently diagnosed female cancer worldwide, with 570,000 new cases occurring in 2018, accounting for 6.6% of all female cancer cases.^(19)^ Concurrent chemo-radiation therapy is the current standard of care for patients with locally advanced cervical cancer, and targeted therapies using tyrosine kinase inhibitors have been consistently attempted to manage metastatic or recurrent patients with low response rates.^(20)^ Even though this disease is generally preventable, the overall prognosis for women with metastatic or recurrent types is poor.^(21)^ Here, we shed light on the *in vitro* anti-cancer activity of NC for treatment of human cervical cancer. NC inhibited cell viability and induced apoptosis in two types of cervical cancer cell lines, Hep-2 and KB (Fig. 1). This result suggests that NC, a naturally derived substance, exhibits anticancer activity in cervical cancer by causing apoptosis.

We used human phospho-kinase proteome profiling to identify the specific protein kinases that play a role in the apoptotic action of NC in human cervical cancer cell lines. The results revealed that phosphorylation of Chk2 at Thr68 is an important regulator of caspase-mediated apoptosis during NC treatment (Fig. 2). The serine/threonine kinase Chk2 is a key component that regulates an appropriate cellular response to DNA damage by phosphorylating substrates involved in DNA repair, cell cycle regulation, apoptosis, and p53 signaling.^(11)^ In particular, p53 appears to be dependent on Chk2.^(22)^ In the present study, we confirmed that p53 accumulation increased rapidly beginning 6 h after NC treatment (Fig. 3). A previous report indicated that p53 is also phosphorylated through Chk2 activation at several individual residues of the N-terminal or C-terminal domain, which is partially required for p53 acetylation in response to DNA damage.^(23)^ Other cumulative data have shown that Chk2 activation leads to p53 stabilization and accumulation through mitigation of p53 degradation by MDM2/MDMX, eventually allowing p53 to bind to numerous target gene promoters.^(24–26)^ Thus, p-Chk2 (Thr68) stimulated by NC treatment could contribute to p53 accumulation. p53 can act as a transcription factor that regulates the proapoptotic Bcl-2 family of genes, including Bim, Bax, and Puma.^(27–30)^ Therefore, we further investigated whether p53 accumulation leads to mitochondria-dependent apoptosis in cervical cancer cell lines. NC treatment significantly altered the expression level of Bim (Fig. 3). Consistent with our data, Han *et al.*^(31)^ also demonstrated that the induced expression of p53 was highly associated with an increased level of Bim, resulting in the release of Bim from sequestration by anti-apoptotic Bcl-2 family proteins. Thus, NC could regulate the p53/Bim signaling axis in its apoptotic activity. Previously, several studies have reported that NC can act as a potent STAT3 signaling inhibitor.^(6,7)^ Based on the results of human phospho-kinase proteome profiling, NC did not affect STAT3 signaling in the KB cell line meaning that it was not commonly related to STAT3 signaling during NC-mediated apoptosis in human cervical cancer cell lines. However, further studies are needed in the future to determine the reason why NC has differential responses on STAT3 signaling in both cell lines because NC still decreases p-STAT3 (Y705) only in the HE-p2 cell line.

Mitochondria, energy-producing organelles, play a central role in the intrinsic apoptosis pathway. Mitochondrial dysfunction such as mitochondrial membrane depolarization triggers the release of an apoptogenic factor (for example, cytochrome c) that participates in caspase-dependent apoptosis.^(32)^ Bcl-2 family proteins can cause mitochondrial outer membrane permeabilization to induce apoptosis.^(33)^ Because we found that NC modulates the p53/Bim signaling axis, we investigated whether NC can affect mitochondrial membrane permeabilization. As expected, NC promoted mitochondrial membrane depolarization and subsequent release of cytochrome c into the cytosol (Fig. 4). Taken together, our results demonstrate the involvement of Bim in the mitochondrial response to p53 during NC-mediated apoptosis in human cervical cancer cell lines.

In response to a DNA double-strand break (DSB), the phosphorylation of histone variant H2AX at the Ser139 residue (γH2AX) occurs.^(34)^ Naturally derived compounds play a crucial role in inducing DNA damage through either increased DSBs or reduced DNA repair mechanisms. Safranal causes H2AX phosphorylation in hepatocellular carcinoma cell lines through inhibition of tyrosyl-DNA phosphodiesterase 1, a main contributor to the DNA repair machinery.^(35)^ The new epipolythiodioxopiperazine derivative G226 inhibits the activity of DNA topoisomerase II and concurrently elevates the expression of γH2AX, contributing to its cytotoxic effect on human cancer cell lines.^(36)^ In our previous study, we demonstrated that the apoptotic effect of oridonin in human oral cancer cell lines requires induction of γH2AX.^(37)^ As in those previous studies, we here found that γH2AX level was greatly increased by NC treatment, and that γH2AX was recruited to DNA break sites to form nuclear foci, suggesting that NC treatment could induce a DNA damage response in human cervical cancer cell lines (Fig. 5). DNA topoisomerases are essential nuclear enzymes that control topological DNA errors in replication, transcription, and recombination. They are affected by topoisomerase inhibitors, which induce DNA damage, cell cycle arrest, or apoptosis.^(38)^ Recently, it was reported that topoisomerases could be targets of NC in cancer treatment, suggesting its function as a topoisomerase inhibitor.^(39)^ Thus, we checked the mRNA levels of *topoisomerase 1* and *2*. The results showed that NC inhibit them only in KB cells, but not in HEp-2 cells (Supplemental Fig. 2*). These results cautiously suggest that γH2AX induction by NC might be caused by inhibition of topoisomerases in a cell context-dependent manner.

In conclusion, the present *in vitro* study indicates that NC plays an apoptotic role through Chk2 activation in human cervical cancer. These findings will help us to develop a new therapeutic option for patients with human cervical cancer.

## Abbreviations

ATM: ataxia telangiectasia mutated
Chk2: Checkpoint kinase 2
ΔΨm: mitochondrial membrane potential
NC: nitidine chloride
NF-κB: nuclear factor kappa-B
