# Supplementary material for: A new insight into the apoptotic effect of nitidine chloride targeting Checkpoint kinase 2 in human cervical cancer in vitro
Source: J Clin Biochem Nutr. 2019 Oct 8;65(3):193–202. doi: 10.3164/jcbn.19-28 (PMC6877403; doi:10.3164/jcbn.19-28)
Supplement: Supplemental Figure 1 [file jcbn19-28sf01.pdf]

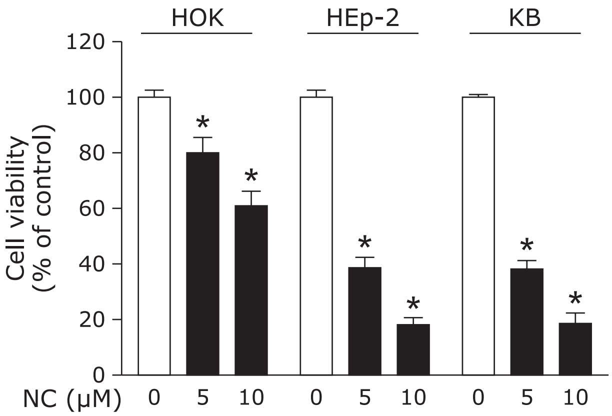

Supplemental Fig. 1. Effect of NC on cell viability in HOK and two cervical cancer cell lines. Each cell line was treated with DMSO or NC. Cell viability was evaluated using a trypan blue exclusion assay. The graphs express the mean ± SD of triplicate experiments (**p*<0.05).
