# Supplementary material for: A new insight into the apoptotic effect of nitidine chloride targeting Checkpoint kinase 2 in human cervical cancer in vitro
Source: J Clin Biochem Nutr. 2019 Oct 8;65(3):193–202. doi: 10.3164/jcbn.19-28 (PMC6877403; doi:10.3164/jcbn.19-28)
Supplement: Supplemental Figure 2 [file jcbn19-28sf02.pdf]

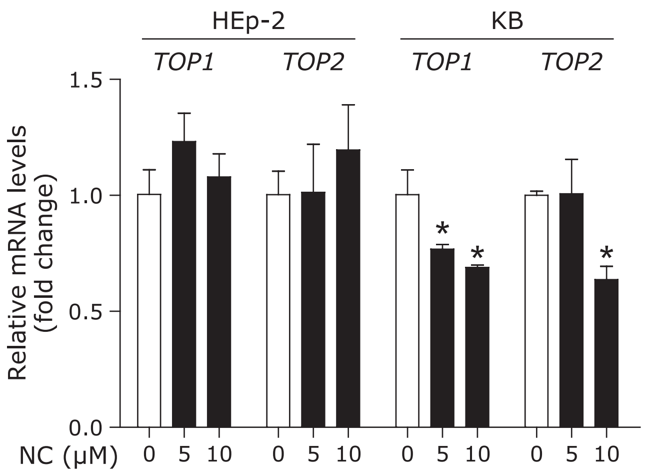

Supplemental Fig. 2. Effect of NC on topoisomerases inhibition in human cervical cancer cell lines. Both cells were treated with DMSO or NC. Expression of topoisomerases was examined by qPCR and expression was normalized to GAPDH.
